# Translational Value of Tumor-Associated Lymphangiogenesis in Cholangiocarcinoma

**DOI:** 10.3390/jpm12071086

**Published:** 2022-06-30

**Authors:** Massimiliano Cadamuro, Adriana Romanzi, Maria Guido, Samantha Sarcognato, Umberto Cillo, Enrico Gringeri, Giacomo Zanus, Mario Strazzabosco, Paolo Simioni, Erica Villa, Luca Fabris

**Affiliations:** 1 Department of Molecular Medicine (DMM), University of Padua, 35122 Padua, Italy; massimiliano.cadamuro@unipd.it; 2 Gastroenterology Unit, Department of Medical Specialties, University of Modena & Reggio Emilia and Modena University-Hospital, 41124 Modena, Italy; adriana.romanzi@unimore.it; 3 Clinical and Experimental Medicine PhD Program, University of Modena and Reggio Emilia, 41124 Modena, Italy; 4 Department of Pathology, Azienda ULSS2 Marca Trevigiana, 31100 Treviso, Italy; maria.guido@unipd.it (M.G.); samantha.sarcognato@aulss2.veneto.it (S.S.); 5 Department of Medicine (DIMED), University of Padua, 35122 Padua, Italy; paolo.simioni@unipd.it; 6 Department of Surgery, Oncology and Gastroenterology (DiSCOG), University of Padua, 35122 Padua, Italy; cillo@unipd.it (U.C.); enrico.gringeri@unipd.it (E.G.); giacomo.zanus@unipd.it (G.Z.); 7 Liver Center, Digestive Disease Section, Department of Internal Medicine, Yale University, New Haven, CT 208056, USA; mario.strazzabosco@yale.edu; 8 General Internal Medicine Unit, Padua University-Hospital, 35122 Padua, Italy

**Keywords:** lymphatic vessel, tumor microenvironment, cancer-associated fibroblasts, biliary neoplasia, VEGF-C, VEGFR-3

## Abstract

The prognosis of cholangiocarcinoma remains poor in spite of the advances in immunotherapy and molecular profiling, which has led to the identification of several targetable genetic alterations. Surgical procedures, including both liver resection and liver transplantation, still represent the treatment with the best curative potential, though the outcomes are significantly compromised by the early development of lymph node metastases. Progression of lymphatic metastasis from the primary tumor to tumor-draining lymph nodes is mediated by tumor-associated lymphangiogenesis, a topic largely overlooked until recently. Recent findings highlight tumor-associated lymphangiogenesis as paradigmatic of the role played by the tumor microenvironment in sustaining cholangiocarcinoma invasiveness and progression. This study reviews the current knowledge about the intercellular signaling and molecular mechanism of tumor-associated lymphangiogenesis in cholangiocarcinoma in the hope of identifying novel therapeutic targets to halt a process that often limits the success of the few available treatments.

## 1. Introduction

Cholangiocarcinoma is the primary liver cancer with the highest lethality, independent of the anatomical subtype, which is currently classified into intrahepatic (iCCA), perihilar (pCCA), and distal (dCCA) [1]. Despite the recent advances in immunotherapy and targeted therapies, the prognosis of CCA remains dismal. Great efforts have been profuse in molecular profiling studies, following the lead of other cancer types, but the identification of actionable genetic mutations (*IDH*, *FGFR2*, and *NTRK*) has not generally resulted in significant therapeutic gains, with a few notable exceptions, e.g., NTRK inhibitors [1,2]. Thus, surgery remains the mainstay of treatment and has the best curative potential for either iCCA, pCCA, or dCCA [3,4].

A recent multicenter study providing a comprehensive analysis of the landscape of CCA in Europe found that surgical resection was performed in 50.3% of patients, with a median overall survival (OS) of 33.4 months (95% CI: 29.1-37.6). Besides the involvement of resection margins, lymph node invasion (N+) was the main factor compromising the OS of patients after resection. Of note, worse outcomes were found in patients with N+ compared to N0 after both R0 or R1 tumor resections (HR = 2.13 (95% CI 1.55–2.94) and HR = 1.61 (95% CI 1.08–2.38), respectively). Furthermore, median OS was affected more by lymph node invasion than by margin involvement, since it decreased from 52.2 months for R0/N0 to 23.3 months for R0/N+, compared with 29.3 months and 21.8 months for R1/N0 and R1/N+, respectively [5]. In line with these findings, an older study from Japan showed that hemi-hepatectomy associated with wide lymph node dissection, extended beyond the regional lymph nodes to the para-aortic site, had curative effects on the 5-year survival in iCCA [6]. Finally, a recent work based on the European Network for the Study of Cholangiocarcinoma (ENS-CCA) registry confirmed that in the case of positive lymph nodes, the state of the resection margin does not influence OS or recurrence-free survival (RFS) [7].

Although resection represents the standard of care, liver transplantation (LT) is considered for both pCCA and iCCA when the tumor is unresectable because of bilateral vascular involvement or because it arises in a background of primary sclerosing cholangitis or cirrhosis [8]. According to the Mayo Clinic protocol, in the staging before LT, lymph node biopsy is an essential step to assess patient eligibility [9]. Unfortunately, lymph node invasion occurs early in the course of the disease, often before the diagnosis of CCA. Lymphatic spread has a deleterious effect on prognosis and precludes any indication for surgical treatments: when lymph node metastases are present at the time of diagnosis, only 2% of patients survive 5 years [10].

As observed in many carcinomas, the dissemination of tumor cells via lymphatic drainage of the tumor represents the most common metastatic route [11,12]. The migration of cancer cells into the lymphatic circulation and spreading to the draining lymph nodes are related to tumor-associated lymphangiogenesis, an intricate and finely tuned process by which new lymphatic vessels are generated from pre-existing conduits and undergo extensive remodeling in conjunction with tumor growth [13]. However, the mechanisms underlying tumor-associated lymphangiogenesis have started to be deciphered only recently. Therefore, strategies able to target lymphatic dissemination are currently lacking, including either traditional chemotherapeutic agents or novel molecules. This review aims at elucidating the molecular underpinnings and cell types regulating tumor-associated lymphangiogenesis in CCA in order to identify targets of translational value to bridge this gap.

## 2. The Lymphatic System in the Liver

The liver is the largest lymph-producing organ, accounting for nearly half of the body’s lymphatic fluid; thus, its lymphatic vascular system is particularly developed compared to other organs [14]. Quite surprisingly, the mechanisms underlying lymph formation and lymphangiogenesis, as well as details on the morphological structure, have been scarcely investigated so far.

Hepatic lymphatic vessels are multifunctional structures: they work as a tissue drainage system, regulating fluid homeostasis and removing waste products and cells, and as an immunological control system, directing the mobilization and activation of immune cells; in addition, they are also involved in lipid metabolism, as they transport lipids to lymph nodes [14].

The lymphatic vascular system is composed of lymphatic capillaries, also called initial lymphatics, and collecting lymphatic vessels [15,16,17]. In the liver, lymphatic vessels are located in three regions: portal, hepatic venous, and sub-capsular areas. The production of hepatic lymph originates in the hepatic sinusoids, filters into the perisinusoidal space of Disse, and enters the interstitial space of portal tracts. Portal lymphatic vessels are the primary site of hepatic lymph drainage, accounting for 80% of hepatic lymph. The rest of the lymph diffuses from the space of Disse into the interstitium around the central vein or under Glisson’s capsule. Portal lymphatic capillaries merge into collecting lymphatic vessels that are surrounded by contractile muscle cells and show continuous “zipper-like” junctions, like blood vessels. Hepatic collecting lymphatic vessels drain fluid into regional lymph nodes in the hepatic hilum [14,15,16,17,18]. From these nodes, lymphatic fluid continues to celiac lymph nodes, eventually draining into the cisterna chili at the lower end of the thoracic duct. Hepatic venous capillaries run along the hepatic vein, merge into collecting vessels, and pass through the diaphragm, together with the inferior vena cava, to mediastinal lymph nodes. Lymphatic vessels underneath the liver capsule drain fluid into diaphragmatic lymph nodes in the thorax and eventually into mediastinal nodes [15,17,18].

Lymphatic capillaries consist of a single layer of lymphatic endothelial cells (LECs) without any smooth muscle cell/pericyte coverage. LECs have discontinuous “button-like” junctions, which efficiently regulate the entry of fluid, antigens, and immune cells into lymphatic capillaries [15]. Besides behaving as structural components of the lymphatic vessels, LECs actively drive the mobilization of immune cells and regulate their functions [15]. Moreover, LECs can collect cholesterol carried by high-density lipoprotein (HDL) through the expression of specific scavenger receptors; indeed, dysfunctional LECs have been linked to fatty liver development [19].

A number of phenotypic markers, more or less specific to the lymphatic lineage, have been recently identified (Table 1), prompting studies aimed at a better understanding of LEC involvement in disease conditions. Podoplanin (D2-40) is a membrane glycoprotein of podocytes and is considered a marker for lymphatic vasculature in humans, since it is not expressed by vascular endothelium or hepatocytes, though some stromal cell types (i.e., fibroblasts and macrophages) may upregulate podoplanin in certain disease conditions [15,20]. Other available markers for lymphatic vessel endothelia include lymphatic vessel endothelial hyaluronan receptor 1 (Lyve1), prospero homeobox protein 1 (Prox1), and vascular endothelial growth factor receptor 3 (VEGFR3) [15,20]; however, these markers are not specific since they can also be expressed by blood vessel endothelium and liver cells. The absence of α-smooth muscle actin expression is also helpful in distinguishing lymphatics from blood vessels in portal tracts [15].

## 3. Lymphangiogenesis in Embryonic Development and in Liver Disease

Lymphangiogenesis is the process by which new lymphatic vessels are formed from pre-existing ones, similar to angiogenesis, which naturally occurs during embryogenesis [15,16,21]. In adults, lymphatic vessels remain quiescent in normal conditions, while lymphangiogenesis reactivates only in pathological circumstances, including tissue repair and inflammation and tumor development. Many cytokines and growth factors are involved in lymphangiogenesis, with a central role played by VEGF-C/D and their receptor VEGFR3, particularly in the liver [15,21,22]. The absence of lymphatic vessels is incompatible with life, and individuals with dysfunctional lymphangiogenesis suffer from chronic lymphedema and immune system functional impairments [15,21].

During embryogenesis in humans, lymphatic vessels arise soon after the cardiovascular system, around embryonic weeks 6-7. They originate from specific subpopulations of endothelial cells located in the lateral parts of anterior cardinal veins that sprout laterally to form primordial lymph sacs. The centrifugal sprouting of lymphatic vessels from these sacs, followed by the complex merging, remodeling, and maturation of newborn vessels, finally generates peripheral lymphatic vasculature [20,21]. It seems that lymphatic vessels may partially derive from mesenchymal lymphangioblasts, which share common origins with vascular progenitor cells. Moreover, hematopoietic cells contribute to lymphangiogenesis by providing paracrine factors [21], as also shown in regenerating mouse liver models and different liver diseases [14,15].

The role of lymphangiogenesis has been investigated in different chronic hepatobiliary diseases, as well as in ischemia–reperfusion injury [15,16,23,24,25,26,27]. In fact, the activation of lymphangiogenesis has been found in chronic viral hepatitis, non-alcoholic fatty liver disease, alcohol-associated liver disease, primary biliary cholangitis, and primary sclerosing cholangitis, resulting in an increased number of lymphatic vessels near areas of fibrosis in conjunction with an inflammatory infiltrate [15,16,23,24,25,26]. Moreover, in non-alcoholic steatohepatitis, increased levels of oxidized low-density lipoproteins promote inflammatory signals and reduce Prox1 levels, leading to decreased lymphatic stability and alterations in liver homeostasis [23]. Impaired lymphangiogenesis has also been observed in mouse models of non-alcoholic steatohepatitis associated with obesity [28].

Increased lymphangiogenesis has been correlated with liver fibrosis and the development of cirrhosis in both humans and animal models [15,16]. In cirrhosis with portal hypertension, active lymphangiogenesis and increased lymph production play a key role in ascites formation, and enhanced lymphatic drainage at the meningeal level ameliorates neuroinflammation and hepatic encephalopathy, as demonstrated in both humans and rats [14,15,29].

Furthermore, in the liver, lymphangiogenesis plays an important role in mitigating inflammation in the early stage of orthotopic transplantation, leading to increased long-term survival in recipients. This difference might be partly due to hepatic immune tolerance, a complex process that involves lymphangiogenesis, since it allows tolerant hepatic dendritic cells to reach the lymph nodes and interact with T cells to establish alloimmunity [14,15].

## 4. Tumor-Associated Lymphangiogenesis: Clinical Significance

**Figure 1:**
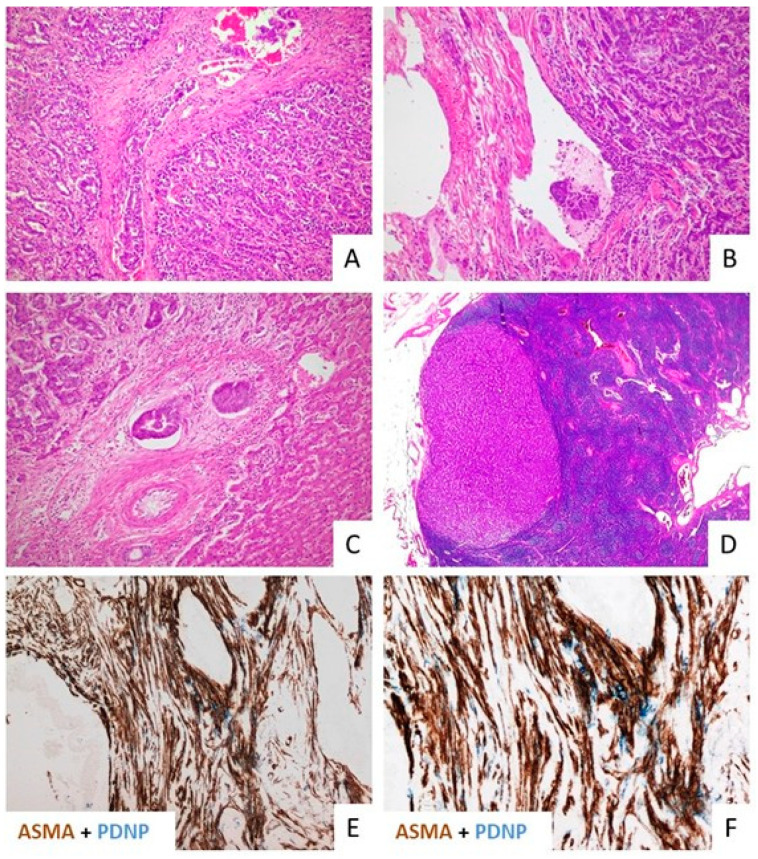
Histological evidence of lymphoinvasion with lymph node metastasis and spatial configuration of the lymphatic vascularization within the tumor microenvironment. (**A**–**D**). H&E showing examples of intra-tumoral (**A**) and peri-tumoral lymphatic vessel invasion in iCCA (**B**); lymphatic invasion can also be observed in portal tracts of adjacent non-tumoral liver (**C**). Neoplastic lympho-invasion in iCCA eventually leads to lymph node metastases (**D**–**F**). Dual immunohistochemistry for Podoplanin (PDPN) (blue) and α-SMA (brown) shows the close alignment of PDPN^+^ lymphatic endothelial cells with α-SMA^+^ cancer-associated fibroblasts in the same area taken at different magnifications, to highlight the intense functional link between the two stromal cell types. Original magnifications: (**A**–**C**,**E**): 10×; (**D**): 1.25×; (**F**): 20×.

The involvement of lymphangiogenesis in the pathogenesis, progression, and response to therapy of malignant tumors is well recognized. The activation of tumor-associated lymphangiogenesis typically occurs in epithelial cancers harboring a prominent stromal reaction in the tumor microenvironment (TME), as observed in pancreatic, breast, prostate, and colorectal cancer, which, akin to CCA, often have a propensity for early lymphatic spread (Figure 1).

The availability of the above-mentioned LEC markers (reported in Table 1) helped to clarify the significance of lymphangiogenesis and its role in metastatic dissemination [30] and enabled the assessment of tumor-associated lymphangiogenesis by quantifying its extent as the lymphatic microvascular density in tissue specimens derived from surgical biopsies. In CCA, extensive lymphatic vascularization (identified by Lyve1) in association with reduced CD34-expressing blood vascularization is a distinctive feature of TME [31].

An abundant lymphatic bed develops in both the peritumoral and intratumoral areas. Although devoid of drainage functions, the newly formed lymphatic vessels are characterized by an increased permeability that favors cancer cell intravasation [32]. However, whether tumor-associated lymphangiogenesis actually provides a way for tumors to disseminate to regional lymph nodes is still a subject of debate [33], but there is accumulating evidence about the notion that increased tumor-associated lymphangiogenesis correlates with the clinical outcome. A high density of peri- and intra-tumoral lymphatic vessels was shown to correlate with increased incidences of intratumoral and peritumoral lymphoinvasion, lymph node metastasis (Figure 1), and tumor recurrence in either pCCA [34] or iCCA [35]. In particular, patients with iCCA and high lymphatic vessel density had a significantly worse OS at 3 years compared with those with low lymphatic vessel density (0 vs. 66.8%) [35]. A similar association was reported in different tumor contexts, such as ovarian [36], gastric [37], bone [38], and breast [39] cancers.

Besides promoting tumor cell dissemination and lymph node metastasis, lymphangiogenesis also plays an important role in the regulation of T cell-mediated anti-tumor immunity, since it promotes naïve T cell infiltration and enhances the anti-tumor effects of immunotherapy, as recently demonstrated in metastatic melanoma and glioblastoma [40]. Understanding if these functions are relevant in CCA represents an area worthy of future research. Unfortunately, to date, there are no treatment regimens that exploit immunotherapy in CCA, although preliminary data from the phase II clinical trial LEAP-05 (NCT03797326) have shown encouraging results in patients treated with the multi-tyrosine kinase inhibitor (TKI) Levatinib in combination with Pembrolizumab, a programmed death-1 immune checkpoint inhibitor [41].

Tumor-associated lymphangiogenesis is finely regulated by combinatorial interactions between different soluble factors released by the tumoral cells themselves and by the multiple cell types populating the TME, such as tumor-associated macrophages (TAMs) and cancer-associated fibroblasts (CAFs). Besides exerting a promoting or inhibitory effect on lymphangiogenesis, cell-derived cues may also contribute to lymphoinvasion. Although studies have been hindered so far by the limited availability of appropriate experimental models and in vitro tools to reproduce tumor-associated lymphangiogenesis, some interesting results have been emerging and hold promise for future translational opportunities. We discuss these concepts in the following chapters.

## 5. Signals Directing Tumor-Associated Lymphangiogenesis and Cell Types Involved

A functional hallmark of CCA is the hypoxic TME, which leads to the increased expression of hypoxia-inducible factor (HIF)-1α, a transcription factor acting as the main effector of hypoxia at the cellular level [42]. HIF-1α is, in fact, able to stimulate the expression of various growth factors, including those of the vascular endothelial growth factor (VEGF) and platelet-derived growth factor (PDGF) families [43]. Interestingly, these growth factors do not work individually but predominantly interact in a complex way through intricate crosstalk, with several signaling pathways involved in both angiogenesis and lymphangiogenesis [44,45].

**Figure 2:**
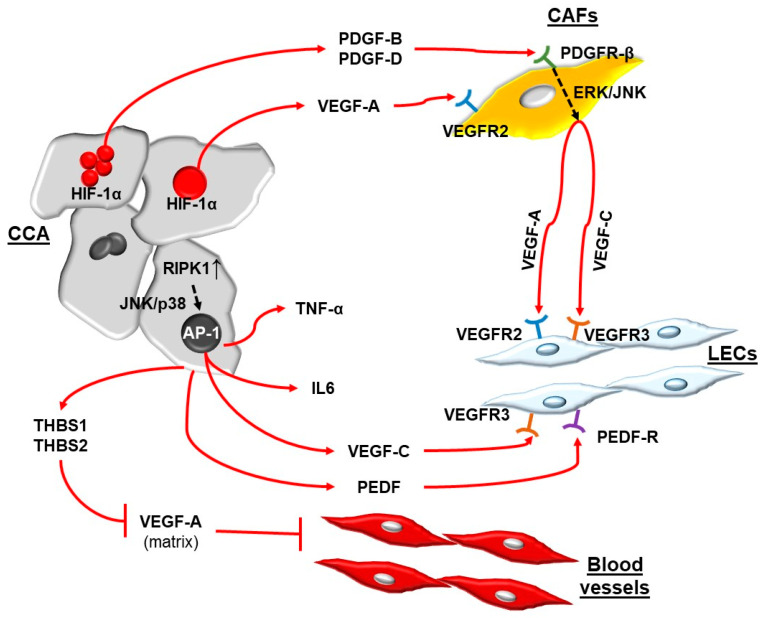
**Molecular mechanisms regulating lymphangiogenesis in CCA.** The recruitment of the lymphatic plexus in CCA is mediated by the coordinated action of neoplastic cells and stromal cells hosted in the tumor microenvironment. Tumor cholangiocytes (CCA), in response to a hypoxic stimulus, upregulate HIF-1α, which is responsible for the increased secretion of VEGF-A, PDGF-B, and PDGF-D. These mediators recruit CAFs, which in turn are induced to secrete VEGF-A and VEGF-C via an ERK/JNK-mediated pathway, ultimately responsible for the vascular assembly of LECs. CCA cells are also able to directly recruit LECs through a RIPK1/p38/JNK/AP-1-mediated pathway that stimulates VEGF-C and PEDF hypersecretion. This same pathway is also able to stimulate the secretion of proinflammatory cytokines such as TNF-α and IL-6, with effects on the inflammatory milieu of the tumor microenvironment. Finally, the secretion of THBS1 and THBS2 by CCA cells, which inhibit the release of VEGF-A by the other components of the tumor microenvironment (matrix), dampens tumor blood angiogenesis in CCA. See the main text for further description of the mechanisms involved. CCA, cholangiocarcinoma; TME, tumor microenvironment; Hif-1α, hypoxia-inducible factor-1α; VEGF, vascular endothelial growth factor; PDGF, platelet-derived growth factor; CAFs, cancer-associated fibroblasts; ERK, extracellular signal-regulated kinase; JNK, c-Jun N-terminal kinase; LECs, lymphatic endothelial cells; RIPK1, receptor-interacting protein kinase 1; AP-1, activation protein-1; PEDF, pigment epithelium-derived factor; THBS, thrombospondin. Legend: ↑, upregulation.

Aishima and coll. showed that in CCA, tumoral bile ducts expressed VEGF-C [46], a member of the VEGF family endowed with the strongest lymphangiogenic properties. By interacting with its cognate receptors VEGFR-2 and VEGFR-3, expressed by LECs [47], VEGF-C is directly responsible for the recruitment of LECs and their gathering in tubular structures. Subsequent studies showed that with a hypoxic stimulus and with the mediation of HIF-1α, malignant cholangiocytes were able to secrete PDGF-B [48] and PDGF-D [31,49]. Both of these growth factors are able to recruit CAFs in close vicinity to the tumoral bile duct mass. In particular, the binding of PDGF-D to its cognate receptor PDGFRβ, expressed by CAFs, triggers a series of biochemical events that sustain tumor-associated lymphangiogenesis and tumor cell dissemination. By activating two distinct pathways dependent on both ERK and JNK, PDGF-D induces CAFs to potently release VEGF-A and VEGF-C, which in turn stimulate LEC accumulation in the TME and their assembly in newly formed vascular conduits abutting tumoral ducts (Figure 2) that are proficient for tumor cell invasion [31].

As mentioned above, in CCA, cells provide an additional source of VEGF-C. This secretory function is stimulated not only by hypoxia but also by the upregulation of receptor-interacting protein kinase 1 (RIPK1). The modulation of this kinase, both in vivo and in vitro, is able to modify the secretion of VEGF-C through the JNK-p38 MAPK-activation protein-1 (AP-1)-mediated pathway. Blocking this pathway yields a twofold inhibitory effect on either the expansion of the lymphatic plexus or tumor proliferation [50]. Moreover, RIPK1 is an upstream regulator of several proinflammatory pathways, including TNF-α, IL-6, and TLR3/4, which is also implicated in tumor-associated lymphangiogenesis in malignant melanoma [51], gallbladder cancer [52], and breast cancer [53]. Of note, increased immunohistochemical expression of RIPK1 in CCA is associated with reduced patient survival [50].

In addition to CAFs and neoplastic cells, TAMs are also capable of VEGF-C secretion. Although data in CCA are lacking, in breast cancer, VEGFR-3-positive TAMs are able to secrete VEGF-C and stimulate tumor lymphangiogenesis and lung metastasis of tumor cells in orthotopically implanted BALB/c and CB.17 SCID mice once selected by treatment with paclitaxel [54]. The importance of the TAM component in the modulation of tumor lymphangiogenesis has been further strengthened by studies in breast cancer models. Very recent papers show that a subset of TAMs expressing another LEC biomarker, i.e., podoplanin, are relevant in lymphangiogenesis and lymphoinvasion. Podoplanin is a membrane glycoprotein constitutively expressed by the lymphatic vasculature, which is instrumental in the proliferation, polarized migration, and tube formation of LECs [55]. Podoplanin-expressing TAMs interact with lymphatic vessels via integrin β1 and β4, thus favoring the increase in the lymphatic bed and metastatic spread. GAL8 expressed by lymphatic vessels mediates binding to integrin β1 when engaged by Podoplanin^+^ TAMs, whereas independently of GAL8 binding, transforming growth factor-β (TGF-β) and various metalloproteinases (MMPs), including MMP2, MMP9, MMP12, and MMP13, overexpressed by TAMs, support lymphangiogenesis and lymphoinvasion via extracellular matrix remodeling [56,57]. Of note, both processes are hampered by deleting *Pdpn* in macrophages [57]. Interestingly, TGF-β has been reported to be a negative regulator of lymphangiogenesis in other cancer types, as shown in mouse xenograft models of pancreatic adenocarcinoma, where inhibition of endogenous TGF-β signaling induced lymphangiogenesis [58]. Although podoplanin has been largely studied in cancer, its role in CCA requires further investigation. Podoplanin is also overexpressed by CAFs and has emerged as an important prognostic marker in pCCA [59]; its overexpression in activated CAFs has been correlated with lymph node metastasis even in iCCA [46].

During the angiogenic processes occurring in both physiological and pathological conditions, VEGF synergistically works with Angiopoietins (Angs). Ang-1 and Ang-2, acting on vascular remodeling through their cognate tyrosine kinase receptor Tie-2, promote the stabilization of newly formed vessels or, alternatively, their destabilization in many types of vascularized tumors [60]. In addition, VEGF and Angs also cooperate during lymphatic vasculature development. Although, unlike VEGF, they are dispensable for the initial development of lymphatic vessels, both Ang-1 and Ang-2 are crucial in the subsequent lymphatic vessel remodeling and maturation, acting as agonist factors, in contrast to what happens during angiogenesis [61,62]. Nonetheless, the expression of the Tie-2 receptor by LECs justifies the relevant function exerted by Angiopoietins on the lymphatic vasculature [63]. Furthermore, all of these angiogenic factors also contribute to tumor-associated lymphangiogenesis and, conceivably, favor tumor cells’ tendency to generate metastasis in solid tumors [64]. As for angiogenesis, it has been shown that Ang-1 potentially has an anti-invasive role, as its expression correlates positively with reduced metastasis in patients with pCCA after surgical resection. Tie-2, expressed by a subset of monocytes, i.e., Tie-2 expressing monocytes (TEMs), correlates with reduced tumor recurrence [65]. In one of the few studies evaluating Ang-2 in CCA, Tang et al. [66] showed that the intensity of Ang-2 expression correlated with the blood microvascular density of the tumor, which, however, appeared less prominent compared to other metastatic tumors.

In line with this observation, and as previously outlined, the relatively scarce blood vascularization observed in the tumoral lesion is likely the flipside of the rich lymphatic plexus featuring CCA. To address this concept, a recent study by Carpino and coll. [67] identified the presence of angio-inhibitory proteins in the extracellular fluid of iCCA cells and found that thrombospondin (THBS) 1 and THBS2, two matricellular proteins, together with pigment epithelium-derived factor (PEDF), induce hypovascularity while promoting lymphangiogenesis. The antiangiogenic activity of THBS1 depends on its inhibition of VEGF-A release from the extracellular matrix by suppressing MMP-9 [68]. CAFs, epithelial tumor cells, and TAMs are all able to variably express these mediators, except for THBS1, which is not expressed by TAMs. However, the pro-lymphangiogenic effects of these proteins seem to be tumor-specific, as THBS1 and PEDF may dampen tumor lymphangiogenesis in colorectal [69] and prostate cancers [70].

Tumor inflammation affects not only cancer cell responses but also LEC biology. In CCA, LECs display an “inflamed” phenotype, enabling them to secrete CXCL5, which acts in a paracrine fashion on tumoral cholangiocytes, inducing the neoexpression of CXCR2, the cognate receptor of CXCL5. The activation of the CXCL5/CXCR2 axis in tumoral cholangiocytes leads to proliferative and cell motility responses associated with the induction of key EMT genes, coupled with perturbed cellular metabolic and bioenergetic activities. Upon CXCR2 stimulation, cancer cells show changes in mitochondrial respiration and in glycolysis, along with the induction of reactive oxygen species, and an increase in glucose uptake and lactate production. In addition, in response to LECs, CCA cells overexpress MMPs, particularly MMP1 and MMP21, resulting in matrix remodeling and further enhancing CCA cell migration and metastasis [71]. Likely, these molecular mechanisms may favor tumor cell invasion of the lymphatic vasculature and subsequent metastasis to regional lymph nodes. It is important to note that LECs’ propensity to develop an “inflamed” phenotype, with the activation of several inflammatory pathways (MCP1, IL1β, IL6, and IL8), is not uncommon, as shown following stimulation with LPS in rat mesentery [72]. Moreover, a lymphangiocrine role of LECs has been recently unveiled during heart development and cardiac repair that is mediated by the extracellular protein reelin, thereby indicating that LECs do not simply serve structural functions to generate vascular conduits but, rather, actively drive paracrine mechanisms regulating tissue growth and injury response [73]. Whether these functions might be exacerbated during malignant transformation is an area ripe for further investigation.

## 6. Therapeutic Opportunities for Targeting Tumor-Associated Lymphangiogenesis

Despite mounting evidence supporting the notion that tumor-associated lymphangiogenesis is an important determinant of outcome in patients with CCA [34,44,46], to date, there are no specific treatments or clinical trials in progress aimed at its targeting. This gap reflects the fact that the molecular mechanisms and cell players involved in this process have started to be elucidated only recently. However, some preclinical studies exploring possible antiangiogenic strategies in CCA are currently ongoing, and on the other hand, data derived from other conditions suggest that drugs already in use may also yield some clinical benefit in CCA. In particular, TKIs, which are already known for their antiangiogenic effects, specific monoclonal antibodies (mAb), or decoy receptors against receptors or ligands involved in tumor lymphangiogenesis and strategies of TME targeting might be considered in this regard (Table 2).

With respect to antiangiogenic drugs, it is important to underline that some of them have shown promising effects not only in the modulation of blood angiogenesis but also in lymphangiogenesis, and therefore, they could be repurposed to counteract lymphatic spread in CCA. However, drugs or molecules able to selectively target the tumor lymphatic vasculature without affecting blood vessels are not currently available for therapeutic use. Sorafenib, a pan-TKI acting on VEGFR2, VEGFR3, PDGFR-β, and Raf-1 and approved for the treatment of advanced HCC, showed scarce efficacy and disappointing results in the treatment of CCA [74]. Another TKI that has proven to be effective in reducing metastatic spread by inhibiting lymphangiogenesis in a preclinical model of colorectal cancer (CRC) was pazopanib, but to date, it has never been tested in a liver setting [75]. Similarly, in a preclinical model of breast cancer, lenvatinib was also able to reduce metastatic spread to either the lung or regional lymph nodes, and this effect was associated with a decrease in the microvascular density of the lymphatic vasculature, consistent with its possible utilization in the treatment of CCA [76]. Furthermore, sunitinib has shown contrasting results on how the pharmacological effects of drugs are dependent on the type and the biology of the primary tumor. In line with these observations, in breast cancer, sunitinib has been effective in countering tumor lymphangiogenesis as well as lymph node invasion [77], whereas in clear cell renal carcinoma, it generated opposite effects [78]. Finally, regorafenib has shown some efficacy both in inhibiting angiogenesis in CCA in clinical trial phase II [79] and in dampening the proliferation of the lymphatic endothelium in mouse models of colon cancer [80].

Currently, monoclonal antibodies or decoy receptors have not been tested in the specific context of CCA in order to inhibit lymphangiogenesis, although some may appear promising based on the results obtained in other pathologies. Among them, bevacizumab, an anti-VEGF-A mAb, approved by the FDA and EMA for the treatment of various cancers, including CRC and HCC, has shown an effect on VEGF-induced lymphangiogenesis in the cornea [81]. Ramucirumab, a mAb against VEGFR-2, was shown to be effective in modulating angiogenesis and metastasis in metastatic CRC [82]. Several mAbs have also been developed against VEGF-C and its VEGFR-3 receptor, but with limited efficacy [90]. Among the anti-VEGF-C mAbs, VGX-100 is in clinical trial phase I for the treatment of glioblastoma, prostate cancer, and metastatic CRC [83], while a single-chain fragment variable (scFv) is still in the preclinical study phase [84]. The mAbs anti-VEGFR-3, IMC-3C5, and small bivalent antibody constructs (diabody) directed against VEGFR-2/VEGFR-3 have been developed and are in clinical trial phase I [85,86], while the fusion protein VEGFR-31-ig and the soluble VEGFR-3 decoy receptor are in the preclinical study phase for the treatment of HCC [87], prostate cancer, and melanoma [88], respectively. However, the effectiveness of these treatments is dampened due to the redundancy of the signals regulating lymphangiogenesis, and thus, targeting other proangiogenic pathways, such as Ang-1 and Ang-2, has been proposed. Among these, trebananib, a neutralizing peptide for Ang-1/Ang-2, is the one in the most advanced study phase (phase III for the treatment of recurrent ovarian cancer) [89], while many other neutralizing mAbs, such as CVX-060, AMG780, and nesvacumab, are in study phase I, but data on their effectiveness are still controversial [90].

Finally, tumor lymphangiogenesis could theoretically be inhibited through manipulation of the TME. There are currently no ongoing clinical trials exploring this approach, but some lines of intervention could potentially be good candidates. For example, given the ability of TAMs to secrete pro-lymphangiogenic mediators, such as VEGF-C [95], one suitable approach could be preventing the recruitment or selectively depleting this cellular component. In preclinical models of mice xenografted with human CCA cells, treatment with 2H5, an anti-MCP-1 mAb [91], and with GW-2580 [92], an inhibitor of the receptor of colony-stimulating factor-1 (CSF-1R), were shown to reduce the recruitment and activation of TAMs. On the other hand, treatment with liposomal-encapsulated clodronate (LIP-CLOD) reduced the number of perilesional macrophages in a xenograft model of CCA [92] and in a genetic model of biliary fibrosis (*Pkhd1^del4/del4^* mouse) [93], whose risk of malignant transformation is known in the orthologous human disease, namely, congenital hepatic fibrosis. CAFs represent another cell population of the TME amenable to intervention to inhibit tumor-associated lymphangiogenesis. Recent studies have shown how treatment with navitoclax, a BH3 mimetic compound, is able to selectively deplete CAFs with a consequent decrease in the lymphatic microvascular density and metastatic spread to the lymph nodes in a preclinical model of syngeneic CCA cell transplantation in rats [31,94].

## 7. Conclusions

We discuss the current evidence on the role of tumor-associated lymphangiogenesis in the clinical progression of CCA, the possible molecular mechanisms, and the multiple cell types involved, with the purpose of identifying putative targets for therapeutic manipulation. Given the lack of treatments able to effectively halt a mechanism that is key in the progression of CCA, we believe that understanding the molecular basis of tumor lymphangiogenesis is a prerequisite for finding new targets that may expand the curative potentialities of the limited treatment options currently available in CCA, surgery in particular. Tumor lymphangiogenesis is paradigmatic of how interactions between cancer cells and the TME may be harnessed for therapeutic gain. New technical approaches, such as deep phenotyping of lymph node metastases, including single-cell transcriptomics, spatial transcriptomics analysis, or tumor organoids, along with the development of appropriate CCA animal models characterized by lymphatic dissemination, will generate novel information critical to enabling the translation to cure. Thus, the time is ripe to include lymphangiogenesis in the equation of CCA progression, prognosis, and treatment response. In this respect, we hope that CCA research may lead the way to future studies that may apply to other epithelial cancers with a propensity for early lymph node metastasis.

## Figures and Tables

**Table 1:** Specific phenotypic markers of lymphatic endothelium.

Marker	Function	Structure
Podoplanin	Mucin-like transmembrane glycoprotein involved in fetal development, platelet aggregation, and migration of T cells and dendritic cells	Transmembrane receptor
VEGFR-3	Cognate receptor for VEGF-C and VEGF-D, involved in normal and tumoral lymphangiogenesis, and in stabilization of lymphatic vessels	Tyrosine kinase receptor
Lyve1	Type I integral membrane glycoprotein, acting as receptor for immobilized and soluble hyaluronan. It is involved in LEC trafficking	Hyaluronan receptor
Prox1	Homeobox transcription factor involved in corneal and lymphatic vessel determination during fetal development, and in stabilization of lymphatic vessels in adults	Transcription factor
Nrp-2	Transmembrane glycoprotein able to bind different ligands. It can act as co-receptor for VEGF-C by binding VEGFR-3	Transmembrane receptor
CCL21	Specifically expressed by LECs, it mediates the trafficking of immune cells (dendritic cells, T cells and neutrophils) expressing its cognate receptor CCR7	CC-chemokine
β-Chemokine receptor D6	Receptor expressed by lymphatic endothelium able to bind several ligands (i.e., MCP-1, MCP-3, MIP-1α)	CC-chemokine receptor
Desmoplakin	Large desmosomal plaque protein involved in cell adhesion due to its bridging action between desmosomes and desmin filaments	Anchor protein
Integrin α9	Heterodimeric integral membrane specifically binding β1 subunit controlling lymphatic valve formation and lymphatic vessel stabilization	Cell adhesion receptor
MRC1	Type I transmembrane receptor binding to L-selectin and involved in trafficking of lymphocytes	L-selectin receptor

**Table 2:** Therapeutic agents of interest for anti-lymphangiogenic strategies in CCA.

Type	Name	Target	Tumor/Disease	Phase	Refs
TKI	Sorafenib	VEGFRs, PDGFRs, c-Kit, RET, BRAF, FGFRs	HCC, CRC, RCC, thyroid cancer, recurrent glioblastoma	Approved	[74]
	Pazopanib	VEGFRs, PDGFRs, c-Kit, FGFRs	Advanced/metastatic RCC, CRC, advanced STS	Approved	[75]
	Lenvatinib	VEGFRs	Thyroid cancer, RCC	Approved	[76]
	Sunitinib	VEGFRs, PDGFRs, c-Kit, RET, CD114, CD135	Pancreatic neuroendocrine tumors, RCC, imatinib-resistant GIST	Approved	[77,78]
	Regorafenib	VEGFRs, TIE2, PDGFR-β, FGFR, KIT, RET, RAF	HCC, RCC, STS, GIST	Approved	[79,80]
Antiangiogenetic mAbs/decoy receptors	Bevacizumab	VEGF-A	Metastatic CRC, breast carcinoma, lung carcinomas, advanced/metastatic RCC, ovarian epithelial carcinoma, primary peritoneal carcinoma, cervix carcinoma	Approved	[81]
	Ramucirumab	VEGFR-2	advanced gastric cancer, gastro-esophageal junction adenocarcinoma	Approved	[82]
	VGX-100	VEGF-C	Advanced solid tumors	Phase I	[83]
	Single chain fragment (scVf)	VEGF-C	Advanced solid tumors	Preclinical	[84]
	IMC-3C5	VEGFR-3	Mesothelioma, thymic carcinoma	Phase II	[85,86]
	VEGFR-31-ig	VEGFR-3	HCC	Preclinical	[87,88]
	Trebananib	Ang-1/Ang-2	Angiosarcoma, ovarian cancer, endometrial cancer, RCC, solid tumors	Phase I	[89]
	CVX-060	Ang-2	Advanced RCC	Phase Ib/II	[90]
	AMG780	Ang-1/Ang-2/Tie-2	Advanced solid tumors	Phase I	[90]
	Nesvacumab	Ang-2	Solid tumors, diabetic macular edema	Phase I	[90]
Other targets	2H5	MCP-1	CCA	Preclincal	[91]
	GW-2580	CSFR1	Neuroinflammation	Preclinical	[92]
	Liposomal clodronate (LIP-CLOD)	Macrophage depletion	CCA, CHF	Preclinical	[92,93]
	Navitoclax	Bcl-2	Lymphomas, advanced solid tumors	Phase I/II	[31,94]

CCA, cholangiocarcinoma; HCC, hepatocellular carcinoma; RCC, renal cell carcinoma; CRC, colorectal cancer; GIST, gastrointestinal stromal tumor; STS, soft tissue sarcoma; CHF, congenital hepatic fibrosis.

## Data Availability

Not applicable.
